# Meckel’s Mystery: Unraveling the Source of Hidden Hemorrhage in a 42-Year-Old Male

**DOI:** 10.7759/cureus.81844

**Published:** 2025-04-07

**Authors:** Martin Nguyen, Samuel Aulick, Savannah Aulick, Marc Subik

**Affiliations:** 1 Radiology, West Virginia School of Osteopathic Medicine, Lewisburg, USA; 2 Clinical Sciences, West Virginia School of Osteopathic Medicine, Lewisburg, USA; 3 Biology, Mt. San Jacinto College, Temecula, USA; 4 Gastroenterology, Joan C. Edwards School of Medicine, Marshall University, Huntington, USA

**Keywords:** meckel´s diverticulum, meckel's diverticulum in adults, meckel's diverticulum management, meckel’s scan, obscure gi bleeding, symptomatic meckel's diverticulum

## Abstract

Meckel’s diverticulum (MD), the most prevalent congenital anomaly of the GI tract, arises from incomplete obliteration of the vitelline duct during embryogenesis. Though often asymptomatic, MD can cause complications like bleeding, obstruction, or inflammation. Diagnosing MD in adults is difficult due to its rarity and symptom overlap with other GI conditions. Modern imaging, particularly the technetium-99m pertechnetate scan (Meckel’s scan), has improved detection capabilities. This case report explores an adult with obscure GI bleeding linked to MD, focusing on diagnostic and therapeutic challenges.

A 42-year-old male presented with a short history of melena and visible red blood in his stools. Initial esophagogastroduodenoscopy (EGD) and colonoscopy at an outside facility failed to identify the bleeding source. Laboratory tests showed severe anemia, prompting a transfusion of packed red blood cells. Despite treatment for a concurrent Clostridium difficile infection, bleeding continued. Further tests, including capsule endoscopy, computed tomography angiography (CTA), mesenteric angiography, and a repeat EGD extending to the jejunum, revealed no clear cause. Due to persistent bleeding, a Meckel’s scan was performed, showing mid-abdominal uptake suggestive of MD. Exploratory laparotomy confirmed MD with mesenteric adhesions, and surgical resection of the affected bowel segment was carried out. The patient recovered smoothly and was discharged post-surgery without issues.

While a frequent cause of GI bleeding in children, MD is often overlooked in adults due to its rarity and vague symptoms, such as bleeding, obstruction, or inflammation. The Meckel’s scan, which identifies ectopic gastric mucosa, is less effective in adults than in children but remains useful. Surgical resection is the standard treatment for symptomatic MD. This case emphasizes the importance of considering MD in the differential diagnosis of unexplained GI bleeding when routine tests are inconclusive. Timely diagnosis and intervention are essential to reduce ongoing blood loss and complications. This report highlights the diagnostic difficulties of obscure GI bleeding in adults and supports a multidisciplinary approach to improve outcomes.

## Introduction

Meckel’s diverticulum (MD) is the most prevalent congenital anomaly of the GI tract, with an estimated prevalence of 0.3%-2.9% in the general population [1,2]. As a true diverticulum, MD encompasses all three layers of the intestinal wall [3] and arises from the incomplete obliteration of the vitelline duct during embryonic development [2,3]. It is more commonly observed in pediatric patients than in adults [4], with most adult cases remaining asymptomatic [5]. Due to its rarity in adults, preoperative misdiagnosis is not uncommon [1]. Advances in diagnostic imaging, particularly the technetium-99m pertechnetate scan (Meckel’s scan (MS)), have significantly improved detection accuracy [1].

We report the case of a 42-year-old male presenting with intermittent dark stools without any obvious source of GI bleeding. An extensive diagnostic evaluation yielded negative results, including two esophagogastroduodenoscopies (EGD), one colonoscopy, one capsule endoscopy, and a selective mesenteric angiogram. Ultimately, an MS was performed, identifying an MD as a probable source of the bleeding.

## Case presentation

A 42-year-old male was admitted to our hospital after presenting with dark stools and visible red blood in his feces for one week, after being transferred from another facility to obtain an Interventional Radiology (IR) consult. Upon his initial examination at the previous hospital, both an esophagogastroduodenoscopy (EGD) and a colonoscopy were performed but failed to identify any bleeding source. His laboratory tests revealed significantly low hemoglobin (Hb) at 6.7 g/dL and hematocrit (Hct) at 19.2% (Table 1), while other vital signs and hematologic parameters remained within normal limits. Consequently, he was prescribed two units of packed RBCs (PRBCs) to address his anemia.

**Table 1:** Initial pertinent laboratory findings at admission. Hb: Hemoglobin; Hct: Hematocrit; PLT: Platelets; MCV: Mean Corpuscular Volume; AST: Aspartate Aminotransferase; ALT: Alanine Aminotransferase; ALP: Alkaline Phosphatase.

Result	Value	Normal Range
WBC	8.6	4-11 (× 10^9^/L)
RBC	2.12	4.3-5.6 (× 10^12^/L)
Hb	6.7	12.1-15.1 g/dL
Hct	19.2	36.1%-44.3%
PLT	228	150-400 (× 10^9^/L)
MCV	90.6	78-100 fL
AST	13	8-33 U/L
ALT	21	4-36 U/L
ALP	43	44-147 IU/L

**Figure 1:**
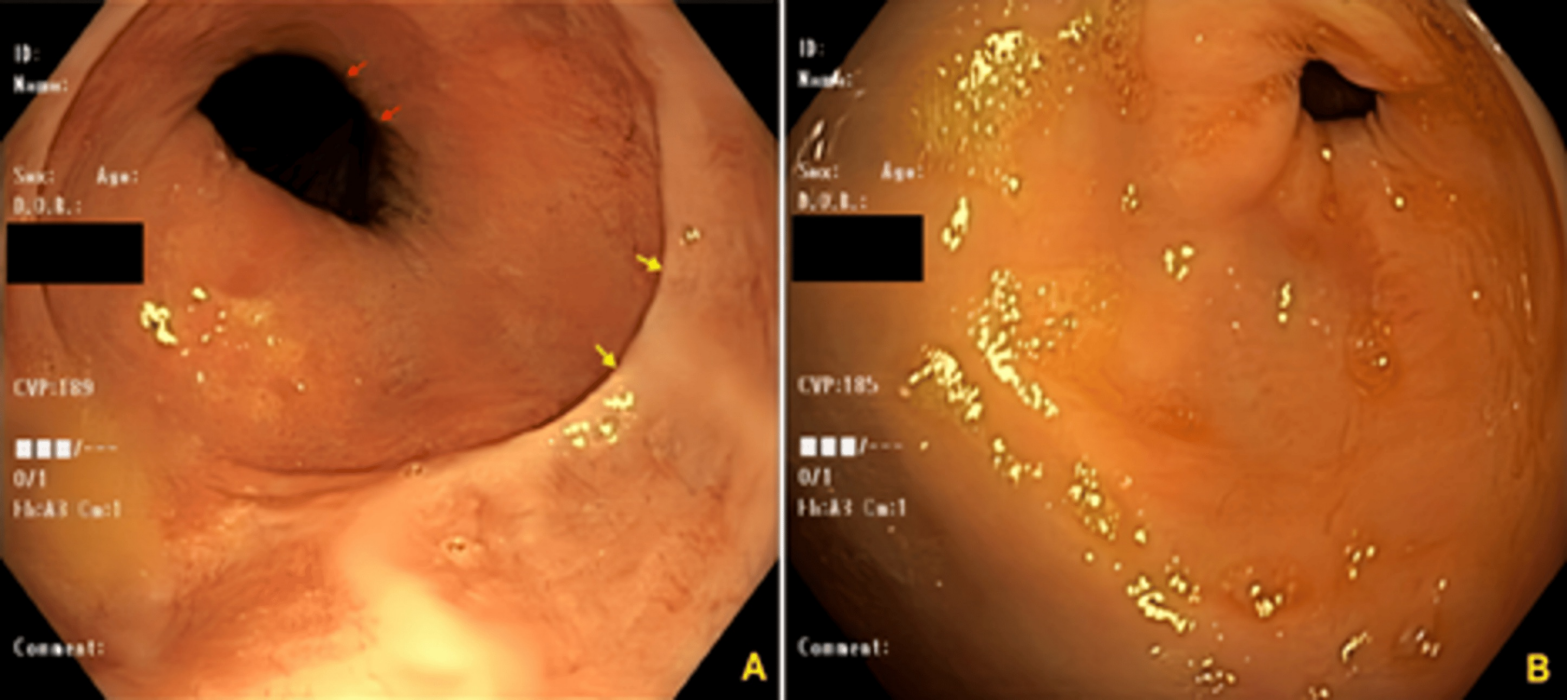
Panel (A), EGD demonstrated a hiatal hernia. However, no active bleeding source was detected. Yellow arrows denote the Z-line (squamocolumnar junction), while red arrows denote diaphragmatic indentation; Panel (B), normal pylorus without any visualized bleeding source. EGD: Esophagogastroduodenoscopy.

**Figure 2:**
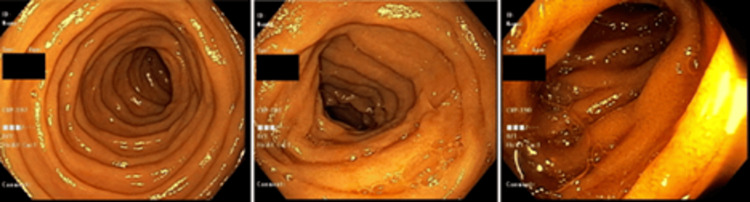
Multiple levels of the duodenum visualized in a repeat EGD failed to demonstrate any active bleeding source. EGD: Esophagogastroduodenoscopy.

**Figure 3:**
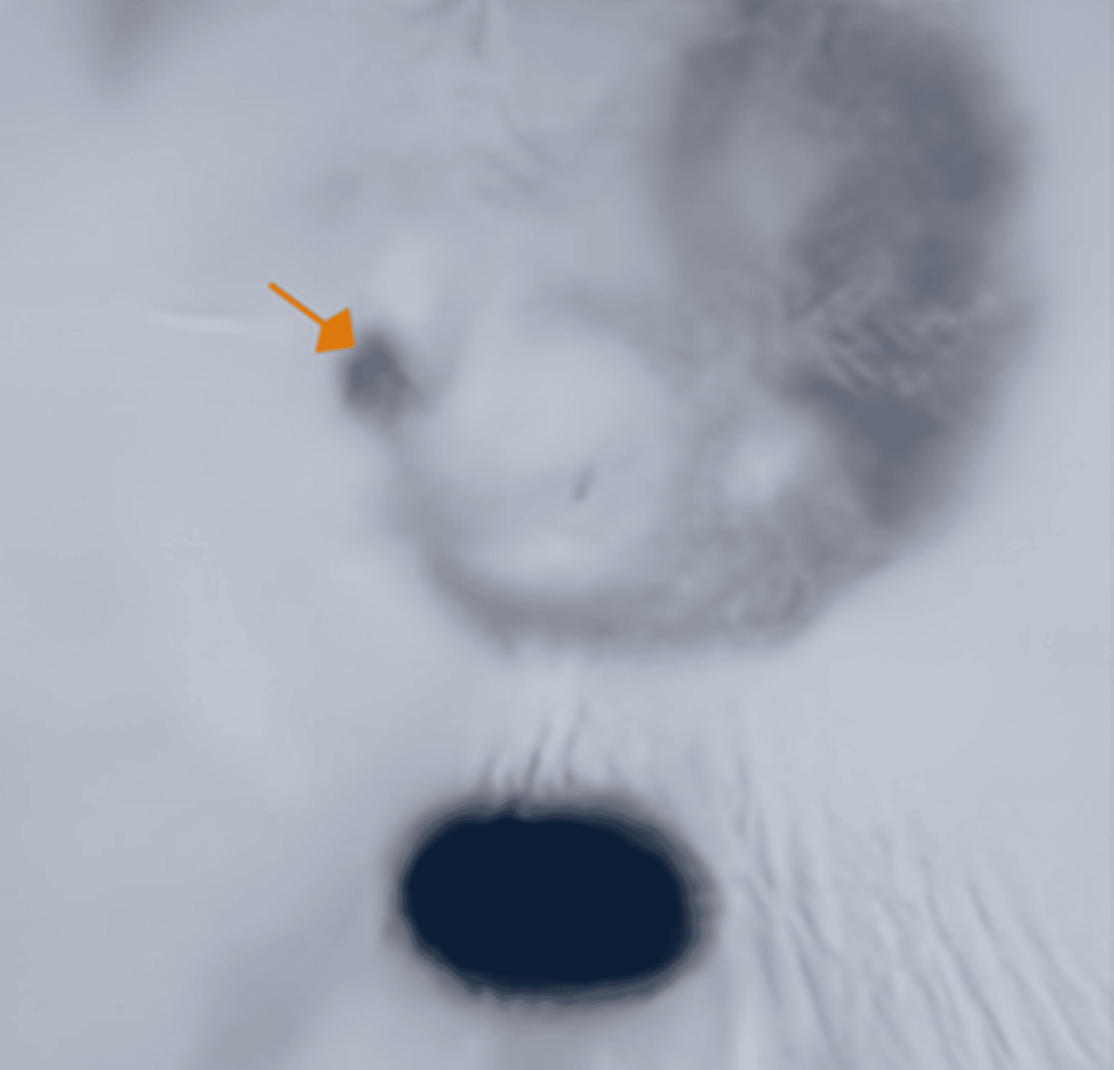
Meckel scan with Technetium-99m demonstrated focused uptake (orange arrow), consistent with a Meckel’s diverticulum.

Further diagnostic efforts included a stool polymerase chain reaction (PCR) which returned positive for Clostridium difficile, leading to the initiation of vancomycin treatment. Despite this intervention, the patient continued to experience dark red stools and GI discomfort. He received Zofran (ondansetron) and pantoprazole (40 mg IV BID) during the same period. A capsule (Pillcam™) endoscopy was performed, which did not reveal pseudomembranous colitis or any evident source of hemorrhage. Given the persistent symptoms and the lack of findings from the initial procedures, a computed tomography angiography (CTA) was ordered but similarly did not pinpoint the source of the hemorrhage. IR was consulted for a mesenteric angiography which came back inconclusive. An additional EGD was attempted, extending through the first third of the jejunum, and there was no evidence of active bleeding (Figures 1-2). A decision was made to perform a Meckel scan. The scan demonstrated a round focus in the mid-abdomen that was suggestive of an MD (Figure 3). Based on this finding, an exploratory laparotomy was deemed necessary. During the surgery, an MD was confirmed to be present in the small bowel with adhesions to the mesentery. The surgical team resected the small bowel segment both proximally and distally to the diverticulum and performed a side-to-side anastomosis using a GI stapler.

Post-operatively, the patient experienced an uneventful recovery. He was discharged five days after the surgery, with no further complications noted during his hospital stay. 

## Discussion

MD is the most common congenital anomaly of the GI tract, resulting from an incomplete obliteration of the vitelline duct during the 6th-10th week of gestation [4]. It occurs in about 2% of the general population [4]. In autopsy series, it was found in 0.14-4.5% of the cadavers [6-8]. The rule of two is often used to describe this condition, which includes the following: (1) it has an approximate length of 2 inches, (2) it is often found within 2 feet from the ileocecal junction, (3) 2% of the population has MD, (4) it is often found in children under 2 years old, (5) and the male/female ratio is about 2:1 [2,5]. Some authors also suggested another 3-to-1 rule: 75% of symptomatic patients were older than 10 years, 75% of symptomatic patients were males, and 75% of bleeding MD contained ectopic gastric tissue [3]. MD contains ectopic or abnormal tissues in 29% of cases, most commonly gastric mucosa [3].

Most MD cases are asymptomatic in adults [5]. In a retrospective study including 1476 patients with MD from 1950 to 2002 at Mayo Clinic, 16% of the patients were reported to be symptomatic [3]. Among the symptomatic cases, male/female ratios were 3:1 [3]. The features associated with symptomatic MD included the presence of histologically abnormal tissue (OR: 13.9; P < 0.001), age < 50 (OR: 3.5; P < 0.001), diverticulum length > 2 cm (OR: 2.2; P = 0.02), and male sex (OR: 1.8; P < 0.001) [3]. Clinical symptoms vary depending on the associated complications of MD (Table 2) [9]. The most common presentation of MD was obstruction (40%) in patients < 11 years of age, and bleeding (38%) in patients older than 11 years [3]. Other common symptoms in adults with MD included obstruction (34%), and diverticulitis (28%) [3]. In the series by Yamaguchi M et al. [9] including 600 patients with ages ranging from 1 day to 91 years, the most common complication of MD included obstruction (36.5%), intussusception (13.7%), inflammation (12.7%), and hemorrhage (11.8%). Bleeding in MD often involves the ulceration of nearby ileal mucosa caused by acid-secreting ectopic gastric tissue [4]. For occult or intermittent hemorrhage, arteriography can be utilized to detect the origin of bleeding [4]. In our case, the patient was indicated for an arteriogram after two inconclusive EGDs. However, it did not detect any source of abnormal bleeding.

**Table 2:** Etiology and clinical symptoms of symptomatic Meckel’s diverticulum in adults. Sources: [10–12]

Study	Total number of adult patients	Number of symptomatic patients	Hemorrhage (%)	Obstruction (%)	Inflammation (%)
Chen JJ et al. [10]	44	21	4.8	28.6	66.7
Zulfikaroglu B et al. [11]	76	36	2.8	66.7	―
Groebli Y et al. [12]	119	52	15.4	23.1	40.4

The lifetime risk of complications from MD ranges from 4% to 6.4% [4,13]. These complications are most common in children under two years of age [3,4,13,14]. The risk then drops significantly to about 1% by age 40 and continues to decline with advancing age, approaching nearly 0% by age 70 [7,14].

In a series of 50 patients with MD, Moore T and Johnston AO [15] reported that 40% of patients with MD had a preoperative diagnosis of acute appendicitis. However, among the cases with a preoperative diagnosis of MD, only 5.7% of those cases had a confirmed MD in the operating room [9]. For bleeding cases of MD, common symptoms include melena and abdominal pain in adults [5]. A total of 90% of these cases contain heterotopic mucosa with gastric mucosa being the most common type [9,16]. The symptomatic gastric mucosa allows 99m Tc-pertechnetate (99m-Tc) to accumulate and become detectable on MS [5,17]. Ectopic gastric tissue is present in 24.2% to 71% of symptomatic MD cases [2]. In the present case, gastric mucosa was identified within the MD specimen. MS is designed to detect the region containing gastric mucosa measuring at least 1.8 cm^2^ [18]. For infants with suspected GI bleeding, MS is recommended to detect gastric mucosa with a high sensitivity and specificity (85% and 95%, respectively). However, MS has a lower sensitivity (60%) in adults [19], together with a positive predictive value (PPV) and negative predictive value (NPV) of 60% and 75%, respectively [19]. Currently, the gold standard for MD diagnosis is surgical exploration [20], as other diagnostic imaging modalities often yield inconclusive results, especially in uncomplicated cases [4]. Sonography is seemingly ineffective when used on adults, although it is still utilized in the pediatric population to avoid radiation exposure. It has higher sensitivity in cases of complications [21]. Regarding barium studies, enteroclysis was shown to be more reliable than conventional techniques for detecting MD [22]. In a series of 415 enteroclyses conducted over 30 months, Maglinte DD et al. demonstrated it correctly diagnosed 11 cases preoperatively (84.6%) among 13 cases surgically confirmed having MD [23]. CT is one of the best imaging modalities in complicated cases in adults such as intra-abdominal abscesses, obstruction, perforation, and tumors [22,24]. In uncomplicated cases, the role of CT is limited because the diverticulum may be mistaken for a small bowel loop [25]. Recently, multidetector CT (MDCT) has significantly increased the sensitivity in MD diagnosis due to the reconstruction power which enables the visualization of the small intestine in multiple planes [26].

The treatment for symptomatic MD is surgical resection [3,4]. For asymptomatic MD in adults, this remains a matter of debate [3,4]. The group favoring abstention from prophylactic removal suggested that the incidence of MD complications decreases with age, thus complications from the surgery itself would potentially be greater than the risks related to the diverticulum [14]. In a retrospective study including 202 MD cases over 15 years, Soltero MJ and Bill AH reported that to save one patient's life from the complications of MD, it would be necessary to remove approximately 800 asymptomatic Meckel's diverticula [14]. The other group which favors prophylactic removal of asymptomatic MD have valid arguments. In a study located in one county, in Minnesota, Cullen JJ et al. [13] reported a lifetime risk of MD complications was 6.4%. Morbidity and mortality in surgical procedures due to MD complications were 12% and 2%, respectively. Meanwhile, the corresponding rates in diverticulectomies for asymptomatic MD were 2% and 1%, respectively. Thus, the authors recommended all the MD discovered incidentally at operation should be removed for most patients, regardless of age [13]. Park JJ et al. [3] also favored the removal of incidental MD which has any of the following features: (1) age younger than 50 years, (2) male sex, (3) diverticulum length greater than 2 cm, and (4) presence of ectopic or abnormal tissue. The complication rate increased when one, two, three, and four of those criteria were reported to be 17%, 25%, 42%, and 70%, respectively [3].

## Conclusions

The rarity of MD and its symptomatic overlap with other acute abdominal conditions often lead to preoperative misdiagnosis. This case underscores that MD is not confined to pediatric patients. Clinicians need to possess a thorough understanding of the embryological origin, clinical manifestations, and radiographic characteristics of this clinical entity. In atypical presentations with occult bleeding, despite extensive diagnostic work-ups, MD should be considered in the differential diagnosis. Early recognition is essential for timely intervention and improved outcomes. While surgical resection remains the definitive treatment for symptomatic MD, optimal management of asymptomatic MD in adults remains uncertain.
